# Brain mechanisms in motor control during reaching movements: Transition of functional connectivity according to movement states

**DOI:** 10.1038/s41598-020-57489-7

**Published:** 2020-01-17

**Authors:** Hong Gi Yeom, June Sic Kim, Chun Kee Chung

**Affiliations:** 1 Department of Electronics Engineering, Chosun University, 309 Pilmundae-ro, Dong-gu, Gwangju 61452 Republic of Korea; 2 Department of Brain and Cognitive Sciences, Seoul National University College of Natural Sciences, 08826 Seoul, Republic of Korea; 3 Interdisciplinary Program in Neuroscience, Seoul National University, 08826 Seoul, Republic of Korea; 4 Department of Neurosurgery, Seoul National University College of Medicine and Hospital, 03080 Seoul, Republic of Korea

**Keywords:** Brain-machine interface, Motor cortex

## Abstract

Understanding how the brain controls movements is a critical issue in neuroscience. The role of brain changes rapidly according to movement states. To elucidate the motor control mechanism of brain, it is essential to investigate the changes in brain network in motor-related regions according to movement states. Therefore, the objective of this study was to investigate the brain network transitions according to movement states. We measured whole brain magnetoencephalography (MEG) signals and extracted source signals in 24 motor-related areas. Functional connectivity and centralities were calculated according to time flow. Our results showed that brain networks differed between states of motor planning and movement. Connectivities between most motor-related areas were increased in the motor-planning state. In contrast, only connectivities with cerebellum and basal ganglia were increased while those of other motor-related areas were decreased during movement. Our results indicate that most processes involved in motor control are completed before movement. Further, brain developed network related to feedback rather than motor decision during movements. Our findings also suggest that neural signals during motor planning might be more predictive than neural signals during movement. They facilitate accurate prediction of movement for brain-machine interfaces and provide insight into brain mechanisms in motor control.

## Introduction

The mechanisms underlying the control of movement by brain are a critical issue in neuroscience^1^. The brain mechanisms have important academic and practical implications. Georgopoulos *et al*. reported that neural signals were altered according to the direction of arm movement^2^. Thus, the trajectories of arm movement can be predicted based on neural signals in monkeys^3–5^ and humans^6–9^. Such prediction of arm movements using neural signals facilitates control of robotic arms by paralyzed patients similar to real arms^10–12^. Therefore, understanding the brain mechanism is of direct interest.

Brain mechanism of motor control has been studied for more than 150 years^13^. Early studies were mainly based on correlations between lesions in the brain and corresponding motor deficits^13^. Results of these studies revealed functional brain areas. Advances in electrophysiological recordings such as electroencephalography (EEG) enable visualization of neural activities occurring in the brain during motor tasks. For instance, the slow negative wave or the Bereitschaftspotential (BP) occurs before the movement generated by primary motor cortex (M1) and supplementary motor area (SMA)^14,15^. Movements induce event-related synchronization (ERS) in delta (0–4 Hz), theta (4–8 Hz) and gamma (above 30 Hz) waves and event-related desynchronization (ERD) in alpha (8–13 Hz) and beta (13–30 Hz) waves in the contralateral motor cortex^16,17^. A beta rebound is generated after the movement^18^. ERD and ERS represent power decrease and increase, respectively. ERD and ERS are generated by a decrease^19,20^ and an increase in synchronous neural populations^21^, respectively. ERD and ERS have been used as the key principles of brain–computer interface (BCI)^22,23^. Since the early 2000s, the mechanism of communication between different brain areas has been actively investigated^24–32^. These studies revealed brain networks during resting state or task performance^33,34^. Especially, studies involving motor functions have analyzed the neural synchrony between M1, SMA, premotor (PM), sensorimotor cortex (SM), parietal cortex and so on in theta (4–8 Hz), alpha, beta and gamma (30–100 Hz) bands^25–30^. The studies revealed that the brain networks differ according to the motor tasks^25,28,30^, types of movement initiation^26^, accuracy of movements^27^ or movement complexity^32^. For example, isometric contraction of the forearm increases brain networks within the beta band between M1 and SMA, whereas repetitive finger movements increases the brain networks within the gamma band and between the theta and the gamma bands from iPM (ipsilateral premotor) to SMA and from iPM to M1^30^. During motor planning, connectivity between ipsilateral SM (iSM) and contralateral SM (cSM) in the alpha band is increased^26^. Rhythmic movements increase beta band coherence between interhemispheric motor areas^31^. Visuomotor tracking tasks increase beta band coherence between motor and visual cortex^31^. Previous studies mainly focused on connectivity during a consistent state^24–32^.

Recently, temporal changes in brain connectivity have been regarded as an important issue because the brain works differently depending on each task^35–37^. Motor control involves interaction between several brain regions^38,39^. Further, the roles of these motor-related regions are changed according to movement states such as perception, motor planning or execution^1,40^. Therefore, it is essential to analyze the changes in motor-related regions according to movement states to elucidate the brain motor control mechanism, which has yet to be investigated. Therefore, the objective of this study was to investigate the brain network transitions according to movement states. In the present study, we measured whole brain magnetoencephalography (MEG) signals in humans during goal-directed reaching movements. Source signals of 24 motor-related areas were extracted from MEG signals using beamforming method. To analyze the changes in brain networks depending on movement states, the functional connectivity between source areas was calculated using mutual information (MI) according to the time window. To reveal the network hubs, the degree centrality was calculated. The aim of this study was to reveal the transition in brain networks by analyzing the transition of connectivities and centralities according to movement states.

## Methods

### Experimental procedure

**Figure 1:**
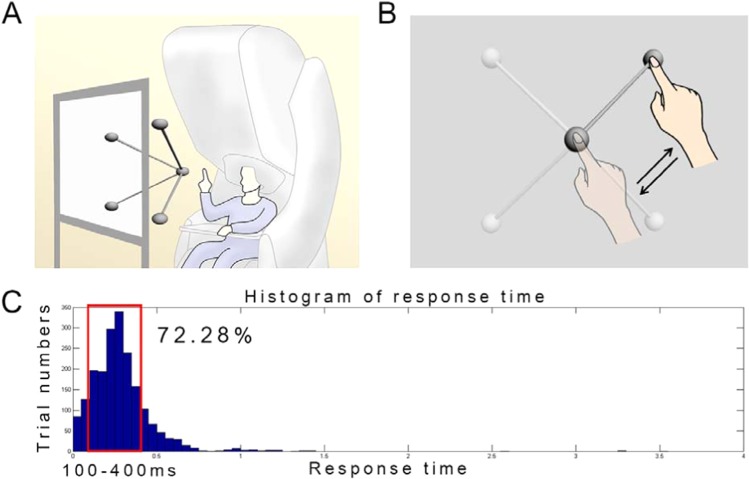
Experimental paradigm. (**A)** Magnetoencephalography (MEG) signal acquisition. Whole-head MEG signals were measured during center-out reaching movements. Stereographic visual stimuli were presented to guide movements. (**B)** Movement tasks. Participants were instructed to move their right arms to reach a target in three-dimensional space according to visual stimuli. (**C)** Histogram of response time. Because large variation in response time altered brain connectivity pattern, trials with response time of 100–400 ms (72.28% of whole trials) were selected for further analysis.

In this study, MEG signals were measured during center-out reaching movements (Fig. 1A). The experiments were performed in accordance with the Declaration of Helsinki and were approved by the Institutional Review Board (IRB) of Seoul National University Hospital (1105-095-363). The experimental procedures were explained prior to the measurements. Informed consent was obtained from all subjects included in the study. Nine right-handed subjects (five males, and four females) aged 19 to 37 years (26.8 ± 6.8, mean $\pm$ SD) participated in this study. The score on the Edinburgh Handedness Inventory was above 80 in all subjects (87.2 ± 5.7)^41^. During the experiment, participants were instructed to move their right arms to reach a target in three-dimensional space according to visual stimuli without any other movements (Fig. 1B). To minimize movement artifacts, a cushion was placed under the participant’s elbow. To present three-dimensional targets, stereographic images were shown on a screen using an STIM2 system (Neuroscan, El Paso, TX, USA). At the beginning of the experiment, a sphere was presented in the middle of the screen for 4 s. During this period, the participants were required to locate their index fingers on the sphere. After the initial period, a target sphere was presented for 1 s in one of the four corners along with a line connected to the target with the center sphere. During this period, the participants were instructed to move their index fingers from the center to the target sphere following the line (a center-out paradigm) and move back to the center sphere. This center-out reaching task was repeated during the session. A total of 120 trials were performed in one session (30 trials in each direction). Two sessions were recorded per subject. Further details of this experiment have been described previously^9^.

### Data acquisition

During the experiment, MEG signals were recorded using a 306-channel whole-head MEG system (VectorView, Elekta Neuromag Oy, Helsinki, Finland) in a magnetically shielded room. The system consisted of 306 sensors in triplets of two planar gradiometers and one magnetometer distributed at 102 locations over the whole brain. The sample frequency of MEG signals was 600.615 Hz. These signals were band-pass filtered at 0.1~200 Hz. Movement trajectories were simultaneously measured using a three-axis accelerometer (KXM52, Kionix, NY, USA) attached to the index finger of the participant with the same sample frequency as MEG signals. We used the spatiotemporal signal space separation (tSSS) method for the MEG signals to reduce the interference from external noise as described previously^42,43^. To align MEG and MRI data, three-dimensional digitization was recorded (FASTRAK, Polhemus, Colchester, VT, USA). All data processing was performed using MATLAB 2018a (Mathworks, Natick, MA, USA).

### Source signal extraction

To examine the activities of motor-related area, the source signals were calculated for 24 regions of interest (ROI). The ROIs related to movements were selected based on references^1,40^. These 24 ROIs included left and right sides of prefrontal area (PF), supplementary motor area (SMA), dorsal premotor (PMd), primary motor cortex (M1), primary sensory cortex (S1), anterior cingulate cortex (ACC), putamen (PUT), pallidum (PAL), thalamus (THA), posterior parietal cortex (PPC), primary visual cortex (V1), and cerebellum (CB). PF is related to movement strategy^40^. SMA and PMd play important roles in motor planning^40^. M1 executes movement of the contralateral body part^40^. S1, V1 and PPC appear to generate the current body position^40^. PUT and PAL receive inputs from the PF, M1 and S1 and provide outputs to the SMA and PMd via THA^40^. PUT and PAL are related to movement initiation^40^. ACC and THA consist of limbic circuits^1^. CB regulates body and limb movements^1^.

MEG and template MRI data were aligned according to three anatomical landmarks (i.e., nasion, left preauricular, and right preauricular). To calculate the source signals from the 24 ROIs, a point in each region was calculated by averaging the Automated Anatomical Labeling (AAL) atlas positions of the region^44^. We created a lead field matrix to describe the relationship between MEG signals and source signals of these points with a spherical head model as described previously^45,46^. If we define *B* as MEG signals, the relationship between MEG signals and source signals can be defined by the following equation:

$$B=A\cdot S+n$$

In the above equation, *S* represents source signals, n denotes measurement noise, and A is the lead field matrix. Because the signal at each point consists of two orthogonal dipole components tangential to the surface of a spherical head model, the source model comprises two source vectors for each point. To calculate the source model, a standardized low-resolution brain electromagnetic tomography (sLORETA) algorithm^47^ was used. *S*_*j*_, the current density at the *j*-th point, is obtained using the following equation:

$${S}_{j}={W}_{j}\cdot B$$

In the above equation, *W* is represents a pseudo-inverse matrix of *A*. Each source location contains two source signals orthogonal to each other. The two signals can be presented as one signal using singular value decomposition, a method used to represent data on a new axis to best describe the data.

### Functional connectivity

To investigate the motor mechanism, we calculated the functional connectivity according to time flow. Source signals were band-pass filtered to low (0.5–8 Hz), alpha (8–13 Hz), beta (13–30 Hz), and gamma (30–200 Hz) frequency bands. The filtered signals of 24 ROIs were temporally segmented based on movement onset. The root-mean-square of the accelerometer signals were calculated and normalized. The time, when the signal began to grow above 5% of a standard deviation, was chosen as the movement onset. The movement onsets were determined by the amplitudes of the accelerometer signals. The movement response time to visual stimuli was 287.0 ± 201.7 ms (mean ± SD). Because large variation in response time altered brain connectivity pattern, trials with a response time of 100 ms to 400 ms (72.28% of whole trials) were selected for further analysis (Fig. 1C). The response time refers to the time between the visual cue and the movement onset. To determine the functional connectivity, the MI between source signals was computed at 10-ms interval from −0.5 s to 1 s based on movement onset with a Hamming window. We selected MI to calculate the connectivity because MI reflects both linear and nonlinear relationships between time series^48^. The window size of MI was 100 ms, which was adequate to calculate MI, although 90 ms of the window was overlapped. Thus, the time interval was 10 ms to analyze rapid changes. MI was then calculated using the following equation:

$$MI({x}_{1},{x}_{2})=\sum _{{x}_{1},{x}_{2}}p({x}_{1},{x}_{2})log\,\frac{p({x}_{1},{x}_{2})}{p({x}_{1})p({x}_{2})\,}$$

where *x*_1_ and *x*_2_ represent 100 ms segments of source signals at 24 ROIs, *p*(*x*_1_) and *p*(*x*_2_) are probability density functions (PDF) of *x*_1_ and *x*_2_, respectively, and *p*(*x*_1_,*x*_2_) denotes joint PDF of *x*_1_ and *x*_2_. These PDFs were calculated from the 100 ms window data of all trials at each time. These MI matrices were then averaged for subjects at each frequency band.

To analyze the communicative ROIs in the motor network, *degree* centralities were calculated by summing MIs connected from a node as shown below:

$${D}_{n}=\sum _{i=n,j\ne n}M{I}_{ij}$$

Centrality is an effective method to represent the sum of connectivity^36^. Thus, it reflects the importance of the node in the network^49^. To highlight the motor network transition, baseline correction was performed by subtracting the mean centrality at baseline. The duration of the baseline ranged from −500 ms to −400 ms. A positive value indicates increased centrality during movement compared with the value during resting state (baseline) while a negative value suggests the opposite.

### K-means clustering

To determine a finite set of motor network, we used the unsupervised clustering algorithm, K-means, for each frequency band^36^. K-means algorithm can be used to separate motor networks into K states. To perform reaching movement, perception, cognition and action are involved^1^. The action includes several processes such as extrinsic kinematics, intrinsic kinematics and kinetics. To examine the change of motor network including these states, we decided to adjust K to 10 to investigate the connectivity change sufficiently. Centralities over time were clustered by using K-means algorithm with nonrandom centroid seeds. To determine centroid seeds, we separated the same 10 intervals and averaged the centralities within each interval. The 10 average centralities were then used as centroid seeds. After clustering, centralities of the same cluster were averaged. As a result, 10 motor networks were determined for each frequency band.

### Visualization of connectivity

**Figure 2:**
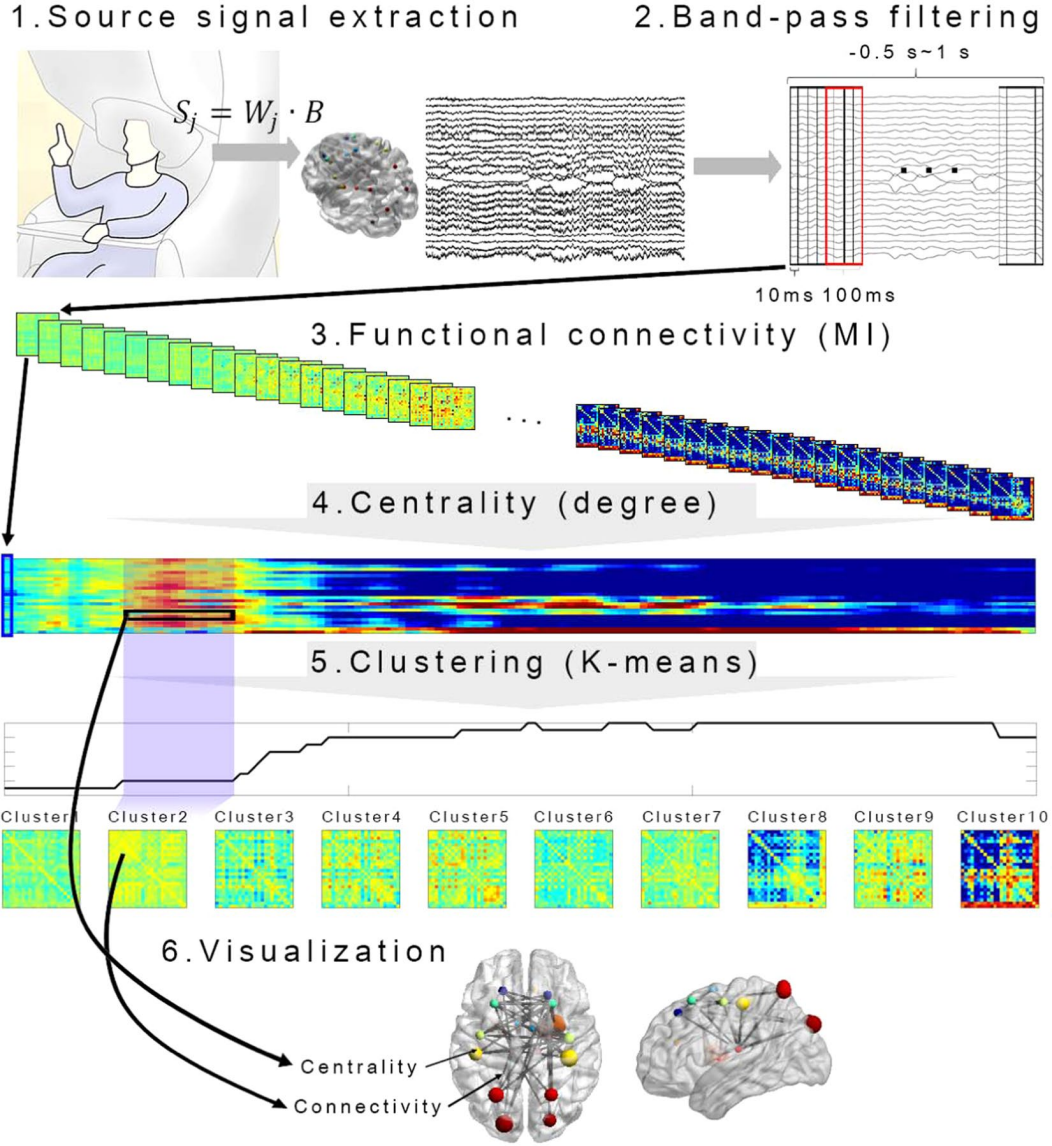
Schematic diagram of data analysis procedures. To examine the activities of motor-related area, the source signals were calculated for 24 regions of interest (ROIs). After band-pass filter and signal segmentation, mutual information (MI) between source signals was computed from −0.5 s to 1 s at 10 ms interval. The window size for MI calculation was 100 ms. To determine the importance of ROIs in the motor network, degree centralities were calculated. To elucidate a finite set of motor network, we used unsupervised clustering algorithm K-means for each frequency band. For intuitive presentation, we demonstrated the connectivity in the brain model using BrainNet Viewer. Connectivity based on MI was defined by the thickness of edge between ROIs. The node size was determined based on centrality.

For intuitive presentation, we illustrated connectivity in the brain model. Connectivity based on MI was represented as thickness of an edge between ROIs. The node size was determined based on centrality. Only connectivity and centrality larger than baseline were described. Therefore, edge thickness and node size indicate the degree of increase in connectivity and centrality according to movement planning and execution. The maximum size of edge or node is limited. To visualize connectivity in a brain model, we used BrainNet Viewer^50^. The data analysis procedure is summarized in Fig. 2.

### Statistical analysis

The aim of this study was to investigate the transition of brain networks according to movement states. Therefore, we assessed the statistical significance underlying the change of centrality before and during the movement compared with baseline. Cluster 2 showed the longest time between clusters before movement. Cluster 2 lasted from −330 to −170 ms before the onset of movement. The duration may be related to the motor-planning state. Duration of the cluster 9–10 was 160 to 500 ms after the movement onset. The duration corresponds to the movement state. Therefore, cluster 2 and cluster 9–10 were selected as a pre-movement state and a state during movement, respectively. Centralities during cluster 2 and cluster 9–10 intervals were compared with the centralities during baselines from −560 ms to −400 ms and from −740 ms to −400 ms, respectively. We used the 160 ms and 340 ms baselines to match the baseline length with the data length during cluster 2 and cluster 9–10 intervals, respectively. Paired-sample t-test with Bonferroni correction was used for statistical analysis using α = 0.001 (n = 48) to compare the centralities in the same brain area.

## Results

**Figure 3:**
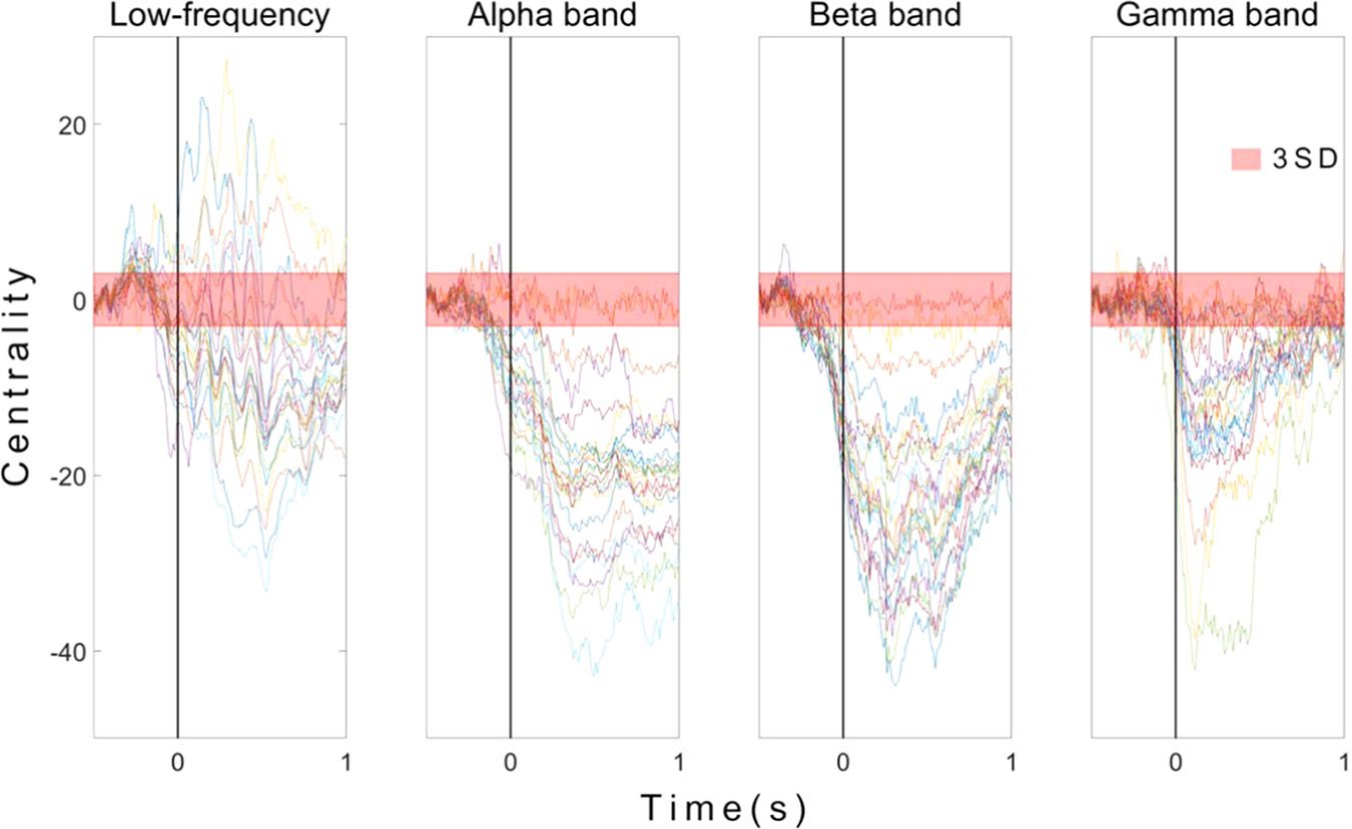
Centralities of 24 ROIs in low-frequency alpha, beta, and gamma bands. Each line represents the centrality of a single ROI. Centralities were normalized by mean and standard deviation (SD) of baseline (−500 ms to −400 ms). Transparent red thick line shows three SDs. The x-axis depicts time in seconds. The y-axis shows centrality in SD units. Time 0 represents the movement onset.

**Figure 4:**
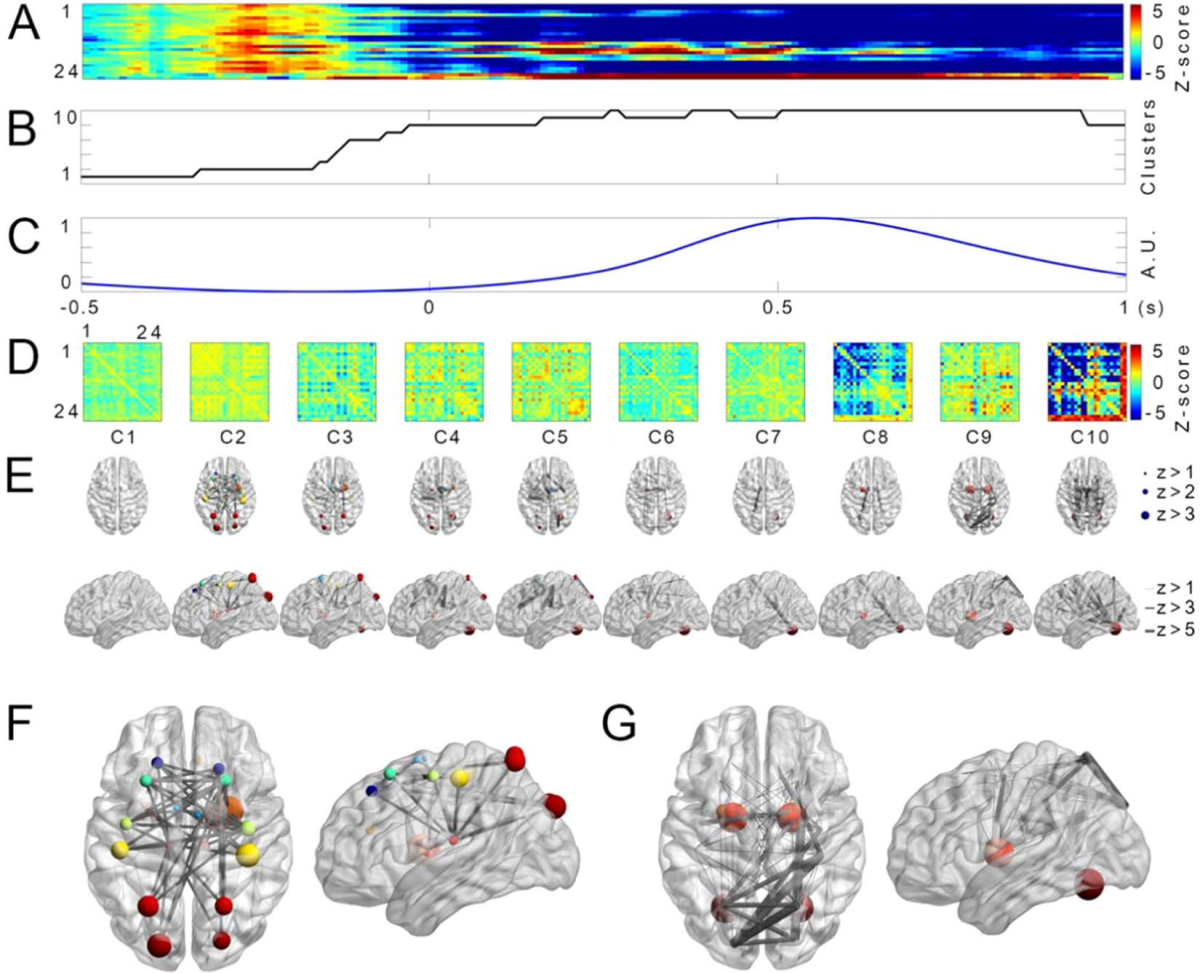
Transition of centralities and connectivities before and during reaching movements in low frequency. (**A)** Transition of centralities of low-frequency band. Colors represent z-scores normalized by centralities during baseline (from −1 s to −500 ms). Red color shows increased centralities. Blue color indicates decreased centralities. The x-axis of (**A**–**C**) denotes time (s). The y-axis depicts 24 ROIs. ROI 1: left PF; 2: right PF; 3: left SMA; 4: right SMA; 5: left PMd; 6: right PMd; 7: left M1; 8: right M1; 9: left S1; 10: right S1; 11: left ACC; 12: right ACC; 13: left PUT; 14: right PUT; 15: left PAL; 16: right PAL; 17: left THA; 18: right THA; 19: left PPC; 20: right PPC; 21: left V1; 22: right V1; 23: left CB; and 24: right CB.(**B)** The cluster number of centralities assigned by the K-means algorithm. The y-axis represents the cluster number. (**C)** Normalized arm position averaged among all subjects and trials. The y-axis represents cluster normalized arm position from center. (**D)** Corresponding connectivities of each cluster number of (**B)**. The x- and y-axes represent 24 ROIs. **(E)** Horizontal and sagittal view of centralities and connectivities in the brain model corresponding to each cluster of (**D)**. Sizes of spheres and lines depict the range of z-scores. (**F)** Centralities and connectivities of cluster 2. (**G)** Centralities and connectivities of cluster 9.

Most centralities were decreased in alpha, beta, and gamma bands in both motor-planning (before 0 ms) and movement states (Fig. 3). In contrast, considerable centralities were increased in low-frequency bands. Centralities of different frequency bands according to time are shown in Fig. 3. Each line represents the centrality of one ROI. Because centralities of bands were increased only at low frequency, the signals at low frequency were analyzed to reveal major regions associated with movements (Fig. 4).

Low-frequency centralities were increased in most motor-related regions in the motor-planning state although most of them were not significant after the Bonferroni correction (Fig. 4). In contrast, low-frequency centralities were increased in CB and basal ganglia (BG) during the movement state. In Fig. 4A,D, colors represent z-scores normalized by centralities at baseline (from −500 ms to −400 ms). The red color shows increased centrality while the blue color indicates decreased centrality. The x-axis denotes time while the y-axis depicts 24 ROIs. Clusters of these centralities are illustrated in Fig. 4B. Figure 4C shows the average arm position among all subjects and trials. The corresponding connectivity of each cluster is shown in Fig. 4D. For intuitive presentation, centrality and connectivities are depicted in the brain model depicted in Fig. 4(E). Sizes of spheres and lines indicate the range of z-score. Large figures of clusters 2 and 9 are presented in Fig. 4F,G, respectively.

**Figure 5:**
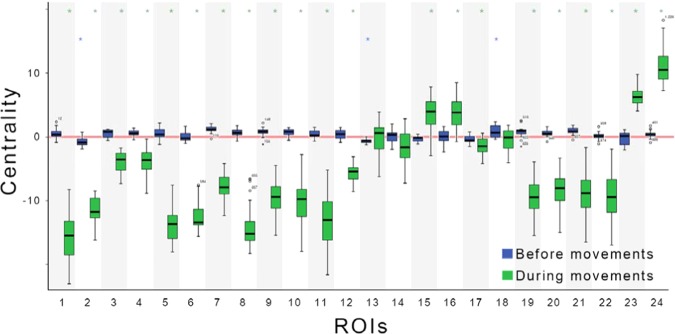
Changes in centralities of 24 ROIs before (from −330 ms to −170 ms corresponding to cluster 2) and during (from 160 ms to 500 ms corresponding to the clusters 9 and 10) movements compared with the baseline values from −560 ms to −400 ms and from −740 ms to −400 ms, respectively. Blue boxes indicate centralities before movements. Green boxes depict centralities during movements. The red line indicates the baseline. Error bars show standard error of mean (SEM). Stars on bars denote significance levels. Statistical significances were calculated by Bonferroni-corrected paired sample *t*-test for each ROI. ROI 1: left PF; 2: right PF; 3: left SMA; 4: right SMA; 5: left PMd; 6: right PMd; 7: left M1; 8: right M1; 9: left S1; 10: right S1; 11: left ACC; 12: right ACC; 13: left PUT; 14: right PUT; 15: left PAL; 16: right PAL; 17: left THA; 18: right THA; 19: left PPC; 20: right PPC; 21: left V1; 22: right V1; 23: left CB; and 24: right CB. *P < 0.05.

Figure 5 shows increase and decrease in centralities during the motor-planning state (from −330 ms to −170 ms corresponding to cluster 2) and movement state (from 160 ms to 500 ms corresponding to clusters 9 and 10) compared with those at baselines from −560 ms to −400 ms and from −740 ms to −400 ms, respectively. Blue boxes illustrate centralities in the motor-planning state whereas green boxes depict centralities during movements. Error bars show standard error of mean (SEM). Stars on bars denote significance level. Most centralities were increased in the motor-planning state except right PF, right PMd, left PUT, left PAL, and left THA (ROI: 2, 6, 13, 15, and 17, respectively). However, centralities on left and right PAL (ROI: 15 and 16) and left and right CB (ROI: 23 and 24) were increased during movement.

## Discussion

### Importance of neural signals during motor planning

**Figure 6:**
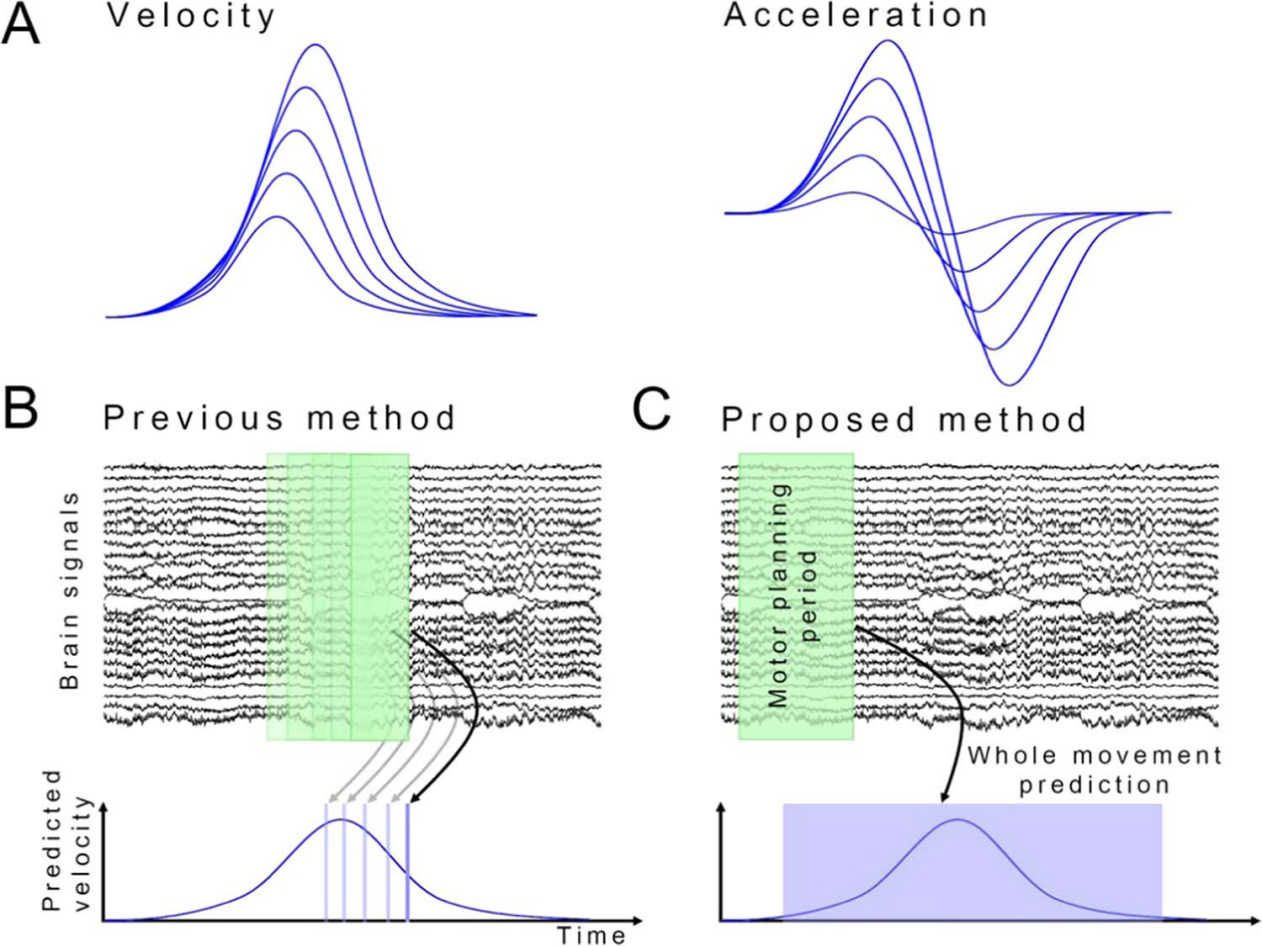
Proposed BMI model for prediction of movement parameters based on brain signals in the motor-planning state. (**A**) Patterns of velocity and accelerometer for different distances. Scale of the pattern is linearly altered according to movement distance. (**B**) Previous method used to decode movement: Previous BMI studies predicted movement velocity using continuous brain signals. (**C**) Proposed method to decode movement: Movement parameters such as reaching distance and velocity were predicted based on brain signals in the motor-planning state.

In general, neural signals carry more information during movement than in motor planning^51,52^. However, our results suggest that most motor-related areas communicated with each other during the motor-planning state, probably related to the Bereitschaftspotential (BP). The increased connectivity can be expressed as a slow wave. The BP is generated from SMA and M1^14^. BP appears to be related to motor planning. During movement, connectivities of CB and BG were increased while those of other motor-related areas were decreased, which may be related to the ERD on the motor cortex. Decreased connectivity can be expressed as desynchronization of neural signals. This decrease does not exclude the role of other motor-related areas during movements. These regions might be involved in movements, albeit with reduced connectivity. These results suggest that most processes involved in motor control might be completed in the motor-planning state. Subsequently, the brain may focus on evaluating the movement to determine differences with previous decisions, suggesting that neural signals in the motor-planning state are more informative about movement parameters compared with neural signals during movements. Previous studies support this view. It has been reported that patterns of speed change during reaching movement are similar, although distances differ^53^. The scale of the pattern is altered linearly according to movement distance (Fig. 6A). Therefore, if the brain works efficiently, it may encode parameters such as reaching distance and maximum velocity instead of continuous speed change. Both the distance and velocity should be determined before movement. Therefore, an increased connectivity during motor planning may suggest processing of information related to reaching distance and velocity.

Compared with motor planning, brain contains a feedback network during movements in which CB and BG are hubs. CB plays an important role in feedback control as an internal model^54,55^. Therefore, damage to the CB causes inaccurate movements^56^. For example, patients with CB lesion may overshoot a target when they make a voluntary movement toward the target^56^ or their hand may oscillate irregularly around the target^1^. BG is also related to motor control. Diseases of the BG lead to various dysfunctions, ranging from hypokinetic disorders such as tremor to hyperkinetic disorders such as chorea^1^. Therefore, brain networks related to feedback are designed to compensate via sensory feedback rather than determination of movement parameters.

In conclusion, neural signals during motor planning are more informative in predicting movements than signals during movements based on our results.

### Implication for brain-machine interface

It has been generally thought that continuous brain waves encode continuous changes in movement velocity (Fig. 6B). Therefore, brain-machine interface (BMI) studies have been performed to decode movement velocity from continuous brain signals^5,10,11,57^. However, our results imply that neural signals during motor planning are more informative than the signals during movements for movement prediction as discussed above. Therefore, we propose to predict movement parameters such as reaching distance and velocity using brain signals in the motor-planning state (Fig. 6C). We expect significant improvements in BMI performance by predicting movements based on motor-planning state.

### Importance of low frequency in motor control

Many connectivities in low-frequency bands were increased under motor-planning and movement states. However, most connectivities in alpha, beta, and gamma bands were decreased or maintained probably due to ERD in alpha and beta. It appears reasonable because the magnitudes of neural signals in alpha and beta were reduced. The connectivity in gamma band can be decreased although the magnitude of gamma signals is increased. These results imply that low frequency plays an important role in motor planning and execution, probably because low-frequency neural oscillations carry substantial movement information such as movement trajectories or velocities^6,9,17,51,58,59^. Our results suggest that movement information is not only processed, but also transmitted in low-frequency oscillation. Because different brain states such as resting, motor planning, and motor execution display different connectivity patterns, brain states can be predicted based on low-frequency connectivity. Such prediction of brain states will be useful for BMI systems.

### Limitations

To investigate motor control mechanism, we analyzed neural signals during center-out reaching movements. The center-out paradigm is a conventional paradigm for the study of reaching movements. Although our results revealed the neural network transition according to movement states, it was difficult to examine the adaptation response to variations such as shifting target position. To investigate the adaptation mechanism, further studies with more complex paradigms are needed. Another limitation of this study is that neural signals originating in deep brain structures have a relatively insignificant effect on MEG signals. Although deep brain signals are weak, source modeling facilitates their investigation^60^. Because it is very difficult to directly measure broad and deep brain signals, studies with source modeling might be the best approach to investigate rapid changes in broad cortical and subcortical brain signals.

## Conclusion

We demonstrated the transition in connectivity between motor-related areas according to movement states. To the best of our knowledge, this is the first study that elucidates the neurophysiology of transition in motor control networks. Our results showed that brain networks differ between motor-planning and movement states. During the motor-planning state, connectivities were increased among most motor-related areas. In contrast, during movement, connectivities of CB and BG were increased whereas those of other motor-related areas were decreased suggesting that most motor control processes might have been completed during the motor planning stage. Further, our results revealed that the brain developed networks related to feedback rather motor decision during movements. Our findings imply that neural signals during motor planning are more informative than neural signals during movement. It provides important insight into the prediction of precise movements for BMIs and elucidation of brain mechanisms of motor control.

## Supplementary information

Supplementary Video 1.

Supplementary Information.

## Data Availability

The data measured and analyzed for the current study are available from the corresponding author on reasonable request.
