# Supplementary material for: Brain mechanisms in motor control during reaching movements: Transition of functional connectivity according to movement states
Source: Sci Rep. 2020 Jan 17;10:567. doi: 10.1038/s41598-020-57489-7 (PMC6969071; doi:10.1038/s41598-020-57489-7)
Supplement: Supplementary file 2 — Supplementary Information. [file 41598_2020_57489_MOESM2_ESM.docx]

**Title**

Brain mechanisms in motor control during reaching movements: Transition of functional connectivity according to movement states

**Author list and affiliations**

Hong Gi Yeom^1^, June Sic Kim^2*^, Chun Kee Chung^2,3,4^

^1^Department of Electronics Engineering, Chosun University, 309 Pilmundae-ro, Dong-gu, Gwangju 61452, Republic of Korea

^2^Department of Brain and Cognitive Sciences, Seoul National University College of Natural Sciences, 08826 Seoul, Republic of Korea

^3^Interdisciplinary Program in Neuroscience, Seoul National University, 08826 Seoul, Republic of Korea.

^4^Department of Neurosurgery, Seoul National University College of Medicine and Hospital, 03080 Seoul, Republic of Korea.

**Corresponding author**

June Sic Kim, Department of Brain and Cognitive Sciences, Seoul National University College of Natural Sciences, 151-742 Seoul, Republic of Korea.

Tel: +82-2-740-8569, E-mail: jskim@hbf.re.kr

**Supplementary Information**

**Video Legend**

**Video 1.** Horizontal and sagittal view of centralities and connectivities on the brain model and corresponding movement position.
